# Alkali Cation Effects on Compressive Strength of Metakaolin–Low-Calcium Fly Ash-Based Geopolymers

**DOI:** 10.3390/ma18174080

**Published:** 2025-08-31

**Authors:** Yan Li, Hongguang Wang

**Affiliations:** 1 China 19th Metallurgical Group Corporation Limited, Panzhihua 617099, China; liyancs@126.com; 2 College of Aerospace and Civil Engineering, Harbin Engineering University, Harbin 150001, China

**Keywords:** geopolymer, fly ash, alkali activator, compressive strength, microstructure analysis

## Abstract

Considering the current requirement for high temperatures and the significant energy consumption in the preparation of geopolymer-based cements, this paper presents a study on the compressive strength of metakaolin-based geopolymers containing various low-calcium fly ash admixtures, prepared at room temperature (25 ± 2 °C). The physical properties and microstructure of the geopolymers were characterized using X-ray diffraction (XRD), Fourier transform infrared spectroscopy (FTIR), scanning electron microscopy (SEM), and energy dispersive X-ray spectroscopy (EDS). The type of alkaline cations, phase transformation, evolution of characteristic functional groups, and hydration characteristics of the microstructures were analyzed, and the hydration mechanism is discussed. The experimental results indicated that the fly ash content had a more significant impact on compressive strength than the alkaline cation type (Na^+^/K^+^). The optimal formulation (20% fly ash with 20% KOH activator) reached a compressive strength of 76.70 MPa at 28 days, which was around 6% higher than that of the NaOH-activated counterpart (72.34 MPa). Crystalline phase analysis in the transformation of mullite and microstructure analysis indicated that the increase in compressive strength could be attributed to the effective filling of the matrix interface by chemically inert fillers and the dense N-A-S-H and C-(A)-S-H multi-dimensional gel structures. These experiments prove the feasibility of using fly ash and metakaolin to prepare geopolymer materials with high compressive strength at room temperature.

## 1. Introduction

Geopolymers are formed when aluminosilicate minerals react with concentrated alkaline solutions. These synthetic aluminosilicate minerals can mitigate environmental issues by reducing the CO_2_ emissions from cement production and use [1,2,3,4]. Geopolymer-based concrete composites exhibit outstanding rheological properties, precise characteristics, low shrinkage, and resistance to fire, acids, alkalis, and ion erosion [5,6,7,8]. Soil stabilization, seawater desalination, hazardous metal ion adsorbent, radioactive waste encapsulation, and geopolymer foam development have all advanced significantly [9,10,11,12,13]. Geopolymer research has gained significant attention due to its promising performance.

Recycled aggregate concrete with a metakaolin geopolymer shows high compressive, tensile, and elastic strength [14]. On the other hand, metakaolin geopolymers, as functional binders, can adsorb heavy metals and methylene blue from water. Due to metakaolin’s non-renewability and its high water consumption in geopolymer production, alternative aluminum-/silica-rich powders are used to substitute it [15]. Several studies have demonstrated that geopolymers can be prepared using powdered coal to obtain good properties [16,17]. The differences in hydration products between the two types of fly ash were thoroughly reported in detail. The use of high-calcium fly ash leads to gel formation [14,18]. CaO in alkaline conditions nucleates gel components, filling interfacial and microstructural gaps [12]. However, when low-calcium fly ash is used, the gels in the system are more typical of geopolymer gels like N-A-S-H (Si/Al ≈ 2, Na/Al ≈ 1), with a small number of other gels present. Moreover, Zhao et al. further classified the type of gels in geopolymer systems by analyzing the relationship between the theoretical value of (Na + K + Ca)/Al and 0.95 [19]. Additionally, the addition of admixtures or functional materials is a common measure to effectively improve the properties of fly ash-based geopolymers. The effect of carbon nanofibers (CNFs) on the microstructure of low- and high-calcium geopolymers was discussed by Zhu et al. [20], emphasizing that CNF agglomeration is more suitable for high-calcium geopolymers, as it improves their mechanical properties and refines their microstructure, than for low-calcium geopolymers, which have lower hydration levels and poor densification. Nematollahi et al. [21] chose naphthalene-based commercial superplasticizers (SPs) as effective activators when using an 8 M NaOH solution. However, when using a multi-compound activator, modified polycarboxylate SP would be better.

The studies mentioned above indicate that, compared to metakaolin, fly ash enhances gels within the geopolymer structure. Additionally, the incorporation of additional additives and functional materials can improve their strength and durability. However, some studies suggest that combining metakaolin and fly ash can further enhance the performance of geopolymers. Bai et al. [22] compared the properties of composites prepared by blending three solid powders of equal mass in metakaolin, including steel slag, brake pad waste, and fly ash. The results showed that the highest compressive strength stability in metakaolin–fly ash geopolymers was observed under various curing conditions. However, lower reactivity and higher packing density in the composites reduced their viscosity and extended the setting time, making them ideal grouting materials for soil reinforcement but not when high early compressive strength is required [23]. To fully utilize fly ash’s potential, Cai et al. proposed increasing the curing temperature to enhance the dissolution of Si and Al from fly ash to achieve higher early compressive strength and a denser structure [24]. Furthermore, it has been reported that the additional introduction of fly ash would considerably improve the fire resistance of metakaolin-based geopolymers [25]. However, the properties of fly ash geopolymer vary; Class F geopolymers are most effective below 500 °C, while Class C geopolymers withstand temperatures above 800 °C, with better thermal reactivity and strength retention. For ideal refractory materials, a higher Si/Al ratio was proposed by Jiang et al. [4] as an important basis for geopolymers to maintain their strength even after high-temperature exposure. However, too high a Si/Al ratio might not be conducive to the development of compressive strength when nano-metakaolin is chosen to partially replace the fly ash powder in the synthesis of geopolymer mortar [26], which would be attributed to the presence of increased unreacted silicate oligomers in the system [27].

Considering that the production of cement requires the combustion of large amounts of fuel, and the decomposition of limestone generates significant CO_2_ emissions, leading to severe environmental pollution, it is crucial to adopt the geopolymer technology to convert waste materials, such as alumina and silica, into high-strength geopolymer concrete. This approach can reduce energy consumption, lower the construction material costs, and minimize the environmental impact of the cement industry. However, available reports on the effect of alkaline cations on the mechanical strength of geopolymers mostly focused on high-temperature environments [28,29], while reports at room temperature remain limited.

Therefore, this study aims to assess the feasibility of the preparation of metakaolin- and fly ash-based geopolymers at ambient temperature (25 °C), analyze the effects of base cations (Na^+^ and K^+^) on the compressive strength characteristics of geopolymers, and characterize the physical behavior and microstructure of geopolymers through microscopic analysis. The hydration sequence and mechanism of the geopolymer system are explained and analyzed. XRD was used to determine the atomic and molecular structure of the material, FTIR was used to identify the functional groups in the compound, SED and EDS were used to observe the microscopic appearance of the sample and perform quantitative and qualitative composition analyses of the material elements.

## 2. Materials and Experiments

### 2.1. Materials

Fly ash and metakaolin from Qiangdong and Jinao were used as precursors. Metakaolin, a common geopolymer precursor, was derived from kaolinite through calcination at 500–800 °C [30]. Fly ash, an industrial byproduct of energy production, has a complex composition and poses potential environmental and health hazards [31]. Additionally, fly ash can be classified into Class F (with low calcium) and Class C (with high calcium) based on its calcium oxide content, with a 10% mass share serving as the threshold. The chemical composition of fly ash and metakaolin, analyzed using X-ray fluorescence spectrometry (XRF), is shown in Table 1.

**Figure 1:**
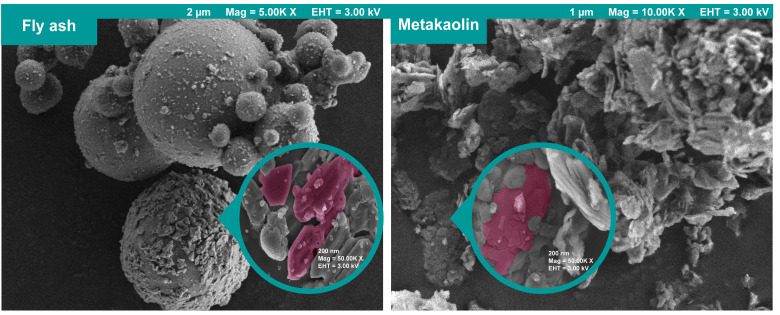
The SEM images of fly ash and metakaolin.

According to the GB/T 50146-2014 standard [32], if the CaO component of fly ash is less than 10%, fly ash is classified as Class F. The morphological images of fly ash and metakaolin, obtained by scanning electron microscopy (SEM) and shown Figure 1, revealed visible spherical particles and a flaky structure, respectively. However, it is noteworthy that there was a great quantity of irregularly shaped amorphous solids that stacked on the surface of the spheres, which is similar to the analysis result of Kutchko et al. [33].

**Figure 2:**
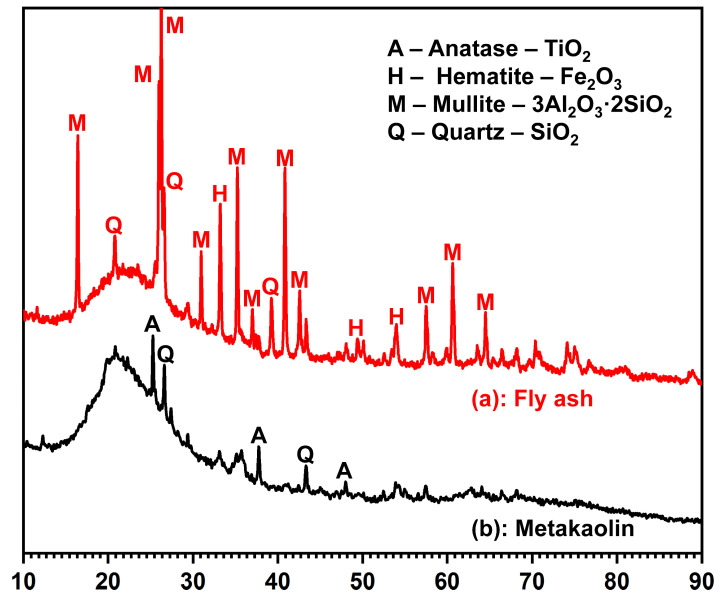
Diagram of the X-ray results for the (**a**) fly ash and (**b**) metakaolin powders.

To further determine the types of solids on the surface of fly ash, Figure 2 presents the X-ray diffraction (XRD) analysis, which revealed significant amounts of mullite (3Al_2_O_3_·2SiO_2_), along with some quartz (SiO_2_) and hematite (Fe_2_O_3_). These findings show that fly ash is a complex mixture of both amorphous and crystalline components [34]. Thus, the formation of these solids may have occurred due to the extensive crystallization of mullite and hematite during calcining [35]. The particle size distribution of the two powders is presented in Table 2.

Two combinations of alkaline exciters were used in this experiment, i.e., NaOH + Na_2_SiO_3_ and KOH + Na_2_SiO_3_. Reagent-grade NaOH granules (96% purity) and KOH granules (85% purity) were supplied by Shanghai National Pharmaceutical Group Chemical Reagent Co. (Shanghai, China). The commercial Na_2_SiO_3_ solution (SiO_2_:Na_2_O:H_2_O = 26%:8.2%:65.8%) was provided by Bengbu Jingcheng Chemical Co. (Bengbu, China).

### 2.2. Preparation of the Activators

To prepare the activators for geopolymer synthesis using two alkaline cations (10 mol/L NaOH and 10 mol/L KOH solutions), reagent-grade NaOH and KOH pellets were dissolved in deionized water. The solutions were then stored in sealed flasks at room temperature (25 °C) for 4 h to ensure complete dissolution and thermodynamic stability before use. Three parallel samples were prepared for each formulation to minimize the experimental error. The mass ratio of Na_2_SiO_3_ to NaOH (or Na_2_SiO_3_-to-KOH ratio) was set at 2.0. It should be noted that the mass of NaOH or KOH granules was calculated based on Equation (1):

*m*_0_ = *cMV*/*w* (1)

where *m*_0_ is the mass of the actually weighed NaOH or KOH granules, *c* is the concentration of the corresponding NaOH and KOH solution (*c* = 10 mol/L), *M* is the relative molecular mass of NaOH (*M*NaOH = 40) and KOH (*M*KOH = 56), *V* is the corresponding volume (1200 mL in this experiment), and *w* is the net content of NaOH granules (*w*NaOH = 96%) or KOH granules (*w*KOH = 85%).

The liquid-to-solid ratio of 0.9 was taken from the authors’ previous analysis result using an orthogonal method [17]. The liquid consisted of the total mass of NaOH + Na_2_SiO_3_ solution or KOH + Na_2_SiO_3_ solution, while the solid was the total mass of metakaolin + fly ash.

### 2.3. Mixing, Casting, and Curing

**Figure 3:**
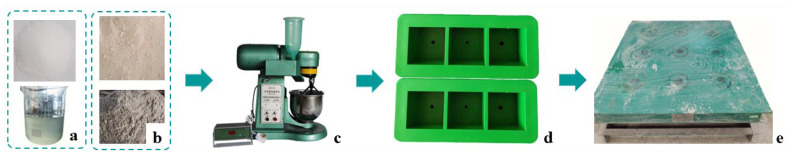
Process diagram for preparing the geopolymer. (**a**) Alkaline activator solution preparation, (**b**) mixing of metakaolin/fly ash powders, (**c**) mixing for slurry homogenization, (**d**) standard 40 mm cubic mold, (**e**) vibration table.

Step 1: Based to the mass data of the powders in Table 3, pure metakaolin powder or a mixed powder of metakaolin and fly ash (see Figure 3b), was firstly stirred slowly for 2 min to ensure its homogeneous mixing. Then the alkaline activator (Figure 3a) was poured into the mixing pot, as shown in Figure 3c, stirring slowly for 3 min, followed by quick stirring for 1 min until the slurry was uniform.

Step 2: The fresh geopolymer paste was poured into a 40 mm × 40 mm × 40 mm plastic mold (see Figure 3d), which had been previously brushed with a concrete release agent. After pouring, the mold was placed on a vibration table, as shown in Figure 3e, and vibrated for about 3 min until no air bubbles appeared on the top surface. To better demonstrate the feasibility of geopolymers in practical engineering applications, all samples were de-molded after 48 h of curing at room temperature. The preparing process is shown in Figure 3.

Step 3: The specimens were cured for 7 days at 25 ± 2 °C and 95 ± 5% humidity before conducting the compressive strength test and other microstructure tests.

The production of samples with a curing age of 28 days followed the same process as described above.

### 2.4. Characterization Methods

As usual, unconfined compressive strength tests were conducted on the 7th and 28th days using a cement compression and flexure testing machine (Hengruijin Testing Machine Co., Ltd., Jinan, China) at a loading speed of 2.4 kN/s. X-ray diffraction (XRD) analysis of the crystalline phase evolution of all geopolymers was performed using a Cu-Kα radiation X-ray diffractometer (D8 Advance, Bruker, Germany) within a diffraction angle (2θ) range of 5–90°, with a step size of 0.0200 and a scanning speed of 5°/min. The FTIR spectra of the geopolymers were recorded using a NICOLET 5700 FTIR spectrometer from Thermo Fisher Nicolet, Waltham, MA, USA. A mixture of potassium bromide (KBr) (100–120 mg) and dried hydrated samples (2–5 mg) was ground for 5–15 min and then compacted under vacuum to form disc-shaped samples. Finally, the test samples were placed in the infrared spectrometer for analysis. The microscopic morphology of the fracture surface of the geopolymers was analyzed by SEM (Regulus 8100, Hitachi, Tokyo, Japan). The incorporated energy-dispersive X-ray (EDS) spectrometer was used to analyze the atomic content without coating. Prior to testing, the samples were fixed on the SEM stage using a conductive adhesive and then gold-coated to enhance their conductivity and ensure the accuracy of the results. The samples were subsequently placed in the electron microscope chamber for analysis.

## 3. Results and Discussion

### 3.1. Compressive Strength

**Figure 4:**
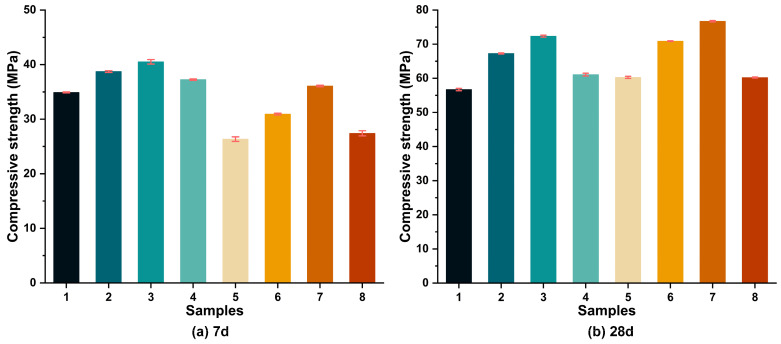
The development of compressive strength from 7 days to 28 days.

The effect of low-calcium fly ash addition on the compressive strength of the metakaolin-based geopolymer with different cation types (Na^+^ and K^+^) is shown in Figure 4. The maximum compressive strength of the geopolymer reached 40.51 MPa at 7 days and 76.7 MPa at 28 days. The addition of fly ash increased the 7-day compressive strength of the geopolymer by 36.94% and the 28-day compressive strength by 27.56%. In general, regardless of the curing cycle (7 days or 28 days), fly ash addition effectively improved the compressive strength compared to the addition of pure metakaolin. It can be seen that the 7-day compressive strength of the geopolymer was found to range from 43.62% to 61.54% of its 28-day compressive strength. All samples exhibited excellent compressive strength during initial curing, likely due to the additional calcium from fly ash accelerating the hydration of the precursor material. Previous studies have reported that Ca^2+^ can accelerate polymerization by providing extra nucleation sites and heat for precipitation [36].

**Figure 5:**
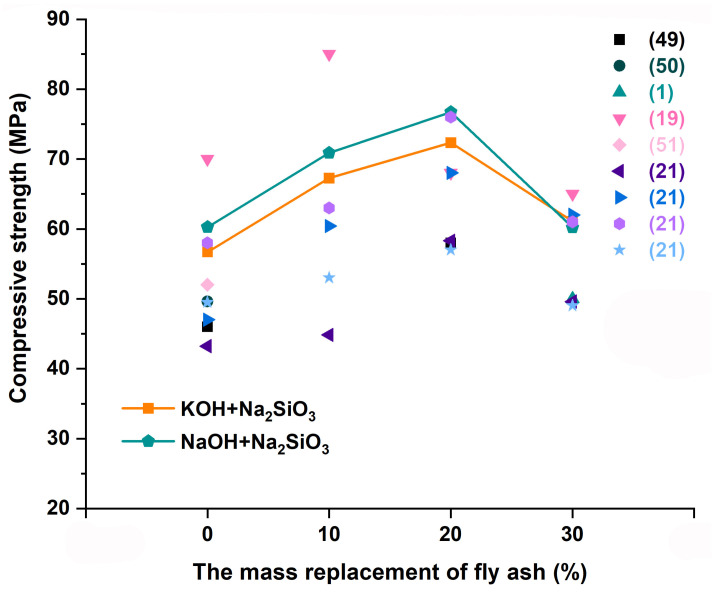
Comparison of 28-day compressive strength.

However, it is clear that no simple linear relationship existed between the content of fly ash and the compressive strength. A slight decrease was observed when the fly ash content exceeded 20%, with the compressive strength decreasing by 24.01% at 7 days and 21.5% at 28 days. The 28-day compressive strength results from this experiment were compared with those by other researchers [3,17,37]. The change trend in compressive strength was similar to that of the test. As the fly ash amount increased, the compressive strength presented a trend of first increasing and then decreasing, as shown in Figure 5.

The higher space-filling capacity of calcium aluminate silicate hydrate particles from fly ash reduced the porosity and enhanced the compressive strength [3]. However, excess fly ash led to the formation of excess calcium silicate hydrate gels in the geopolymer system. Despite the significant increase in compressive strength compared to that observed for pure metakaolin-based polymers, the compressive strength was lower than that of calcium aluminate silicate hydrate and (sodium, potassium) aluminate silicate hydrate gels [38], but still higher than that of pure metakaolin-based geopolymers. In addition, the filling effect provided by the unreacted fly ash particles, when the mass replacement exceeded 20%, provided a worse result due to significant damage to the integrity and uniformity of the microstructure during curing [3]. Furthermore, the compressive strength of all samples significantly improved after 28 days of curing, which was mainly attributed to the continuous hydration of the precursor materials and the densification of the matrix. As the reaction and leaching of Ca^2+^ from fly ash continued, the additional SiO_2_ and Al_2_O_3_ could react and form calcium silicate hydrate/calcium aluminate silicate hydrate and (sodium, potassium) aluminate silicate hydrate gels, leading to a higher strength geopolymer [39]. Li et al. [12] attributed the increase in geopolymer intensity to a continuous polymerization reaction, the formation of calcite, and the promotional effect of calcium. The specific role of calcium in the fly ash used in this experiment, as well as the possible reaction mechanism, will be discussed in detail later.

On the other hand, it can be seen that the early strength of the (NaOH + Na_2_SiO_3_) samples was significantly higher than that of the (KOH + Na_2_SiO_3_) samples, by up to 35.86%, which was mainly attributed to their better capability to release monomers of silicate and aluminate from the precursor materials [40]. Collins et al. also reported that the greater charge density of Na^+^ compared to K^+^ (1.06 vs. 0.59) resulted in faster dissolution of Si and Al in precursor materials [41]. However, it can be clearly seen that the compressive strength of the (KOH + Na_2_SiO_3_) samples was slightly greater than that of the (NaOH + Na_2_SiO_3_) samples at 28 days, with a maximum difference of 6.28%. According to the results of Liew et al. [42], the large oligomers formed by the pairing process of large K^+^ ions with the silicate anion lead to the K-geopolymer having superior compressive strength. The combination of metakaolin and low-calcium fly ash studied in this experiment has been fully reported in previous studies for its excellent machinability, mechanical properties, and durability. However, due to its complex structure and hydration products, it is necessary to explain the geopolymer strength from a microscopic perspective and provide a logical relationship between the hydration system order and the hydration products for its development and practical engineering applications.

### 3.2. Microstructural Analysis

#### 3.2.1. Crystalline Phase Analysis

**Figure 6:**
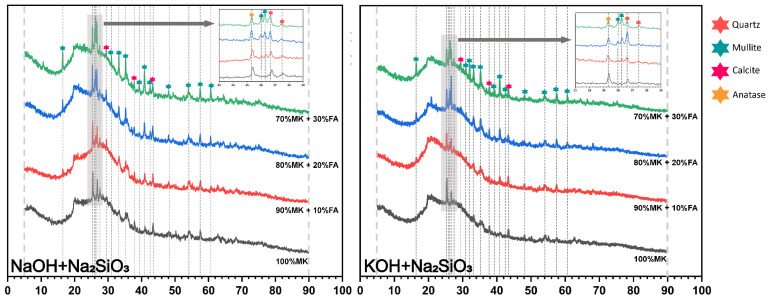
The analysis results of XRD at 7 days.

**Figure 7:**
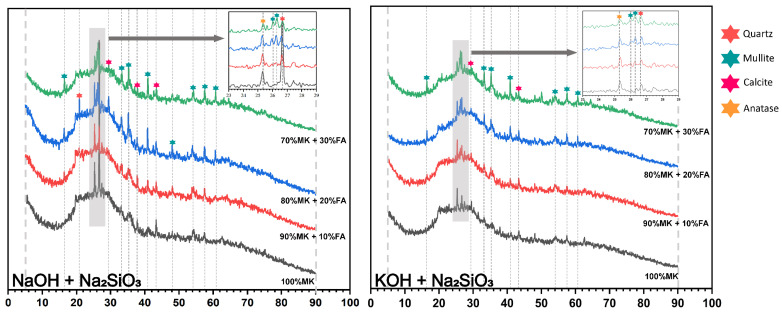
The analysis results of XRD at 28 days.

The phase change and transformation observed from the XRD analysis results contributed to the study of the evolving characteristics of the geopolymer’s microstructure. The XRD spectrum in Figure 6 and Figure 7 shows the dominance of crystalline phases in the solid metakaolin-based geopolymer composites with different fly ash content. Compared to the XRD pattern of the raw materials shown in Figure 2, all samples after the polymerization reaction for 7 days presented broad humps centered at approximately 24–28° (2θ), which corresponded to the reported reaction products of (N, K)-A-S-H and C-(A)-S-H [12], involving the partial hydrolysis of the raw materials and the formation of new phases. Sodium aluminate hydrate (N-A-S-H) gel is the main product formed in the alkali excitation reaction process and is responsible for strength development in polymer materials [43]. In addition, the hydrated calcium aluminate silicate (C-A-S-H) gel is an aluminum-modified hydrated calcium silicate gel, formed as a result of the dissolution and reaction of CaO. In the literature, reaction products such as N-A-S-H and C-A-S-H are commonly described as a ‘featureless humps’ centered at approximately 27–29° (2θ) due to their amorphous and semi-amorphous nature [44,45].

It has been reported that such gels in the form of an intercrossed network could contribute to the improvement of the pore structure in metakaolin-based geopolymers [46]. The characteristic crystalline peaks detected for the geopolymers were associated with quartz (SiO_2_), mullite (3Al_2_O_3_·2SiO_2_), calcite (CaCO_3_), and anatase (TiO_2_). The corresponding diffraction peaks were observed at 26.64°, 26.26°, 27.42°, and 25.28° [47,48,49]. Quartz is a common residual crystalline phase in polymerization systems of geopolymers because of its incomplete solubility in alkaline environments and is presumed to be a potential basic filler able to enhance the mechanical strength of geopolymer products. Notably, the variation in the residual crystalline phase of mullite could reflect numerous key information. The number and sharpness of the characteristic peaks related to mullite increased significantly with the increase in fly ash content, further proving that the main source of mullite was fly ash. In addition, the intensity of the mullite peaks gradually decreased with the curing time from 7 days to 28 days, and then the peaks disappeared or became weaker. This indicated that the silica and aluminum oxide dissolved and formed an amorphous gel product, thereby improving the compressive strength of the geopolymer specimens. However, the intensity of the quartz phases did not show a clear linear relationship with the compressive strength, indicating that when the fly ash admixture exceeded 20%, the elevated chemically inertia of the composition was detrimental to the density and coherence of the matrix, thus reducing its mechanical strength. The crystalline phase of anatase detected after 7 days and 28 days mainly acted as an internal filler to enhance the compressive strength. The appearance of calcite might be due to the alkaline solution containing Ca^2+^ emerging on the surface of the specimens through the pore structure and undergoing a carbonation reaction with carbon dioxide in the air. In addition, the analysis results of the crystalline phase differed from the results of Fu et al., who did not detect any zeolite phase in the samples prepared in their experiment [8], which may be related to the concentration and composites of the alkaline exciter or to the calcium content.

#### 3.2.2. Functional Group Identification

**Figure 8:**
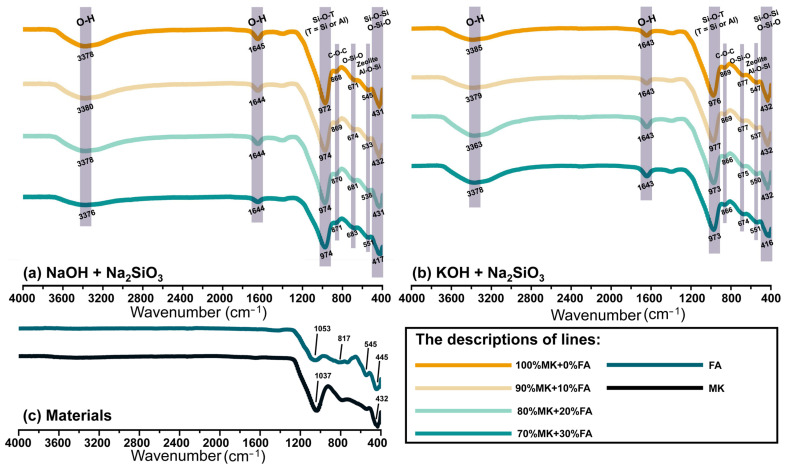
The analysis results of FTIR at 7 days.

**Figure 9:**
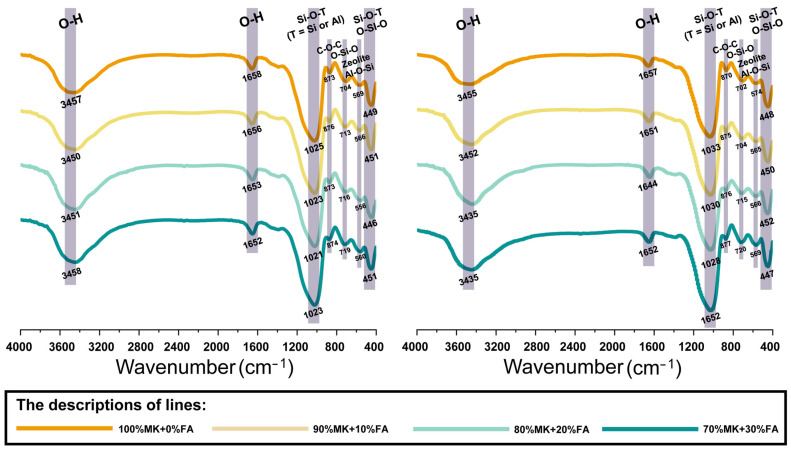
The analysis results of FTIR at 28 days.

The FTIR spectra of fly ash, metakaolin, and the metakaolin-based geopolymers with the different fly ash mass contents are showed in Figure 8 (7 days) and Figure 9 (28 days). For fly ash, the peak at 1053 cm^−1^ is attributed to the stretching vibration of Si-O-Si in clay minerals like mullite [9], while the peak at 817 cm^−1^ is attributed to the symmetric stretching vibration of Si-O-Si bonds and the stretching vibration of Al-O in mullite-like structures [50]. These two bands also supported the presence of mullite crystals, as indicated by the XRD results (Figure 2). The adsorption peak at 535 cm^−1^ is due to the symmetric tensile vibration of Al-O-Si. In the structure of zeolite, the silico-oxygen tetrahedron and alumino-oxygen tetrahedron are connected by a shared oxygen atom, and the symmetric tensile vibration of Al-O-Si is an important vibration mode in the zeolite structure [51]. The band in the range of 430–445 cm^−1^ is due to the flexural vibrations of Si-O-Si and O-Si-O associated with amorphous silica. In zeolites, the interactions between silica tetrahedrons and between silica tetrahedrons and amorphous silica lead to the appearance of flexural vibrations of Si-O-Si and O-Si-O [52]. Zeolites usually show specific absorption peaks in the infrared spectrum, and the vibrations of the Si-O-Si and Si-O-Al bonds exhibit distinct absorption peaks in the infrared spectrum. The size of the zeolite crystal is in the nanometer range, and the smaller the size of the crystal, the wider the diffraction peak, and the weaker the strength. This can make the characteristic peak in the XRD pattern less obvious or undetected. This is mainly because the diffraction of X-rays is limited by the size of a crystal. When the crystal size is close to or less than the X-ray wavelength, the diffraction signal becomes blurred. In the FTIR spectra of metakaolin, the main bands at 1037 cm^−1^ is attributed to the asymmetric vibration of Si-O [53].

After the alkali activation of the composites for 7 days (Figure 8), there was no obvious difference related to the two types of activators. This result is consistent with the findings of Allali et al. who researched the influence of calcium content on the performance of metakaolin-based geopolymers with two types of alkali cations (Na^+^ and K^+^) [54]. The broad peaks observed in the region of 3360–3390 cm^−1^ were attributed to the stretching vibration of O-H, while the peaks that appear near 1640 cm^−1^ correspond to the bending vibration of O-H from the physically adsorbed H_2_O, likely originating from the surface of the geopolymer structure or from imprisoned water in the cavities of the samples [9]. The major vibrations observed around 970–980 cm^−1^ are associated to the formation of asymmetric stretching vibrations of Si-O-T (T = Si or Al) in the three-dimensional geopolymer network, which is characteristic of geopolymer gel formation [55]. Furthermore, the gradual leftward shift in the spectrum of Na-based geopolymers, as the mass ratio of fly ash increased, indicates the formation of more Si-O-T bonds with the addition of fly ash. A similar shift was observed in the spectrum of K-based geopolymers, but without a linear relationship between the mass ratio of fly ash and the degree of the leftward shift. Meanwhile, the vibrations in this range indicate the large presence of synthetic C-S-H-type gels, though a small amount of C-A-S-H gels may be present in the case of alumina [30]. The sharp peaks in the range of 865–870 cm^−1^ correspond to the C-O-C bonds appeared during the formation of calcite (CaCO_3_), when an alkaline solution with Ca^2+^ from the outer surface of the sample reacts with CO_2_ in the air [51,56], further demonstrating the accuracy of the XRD results (Figure 6 and Figure 7). The weak peaks in the region of 670–685 cm^−1^ correspond to O-Si-O bands associated with the quartz species [55,57]. Additionally, the weak peaks in the ranges of 545–555 cm^−1^ and 530–540 cm^−1^ were mainly contributed by the symmetric stretching vibration of Al-O-Si [51]. The peak at 416 cm^−1^ corresponds to the bending vibrations of O-Si-O [58].

After the polymerization process of 28 days (Figure 9), all the bands presented a slight left leftward shift in the range of 0 to 100 cm^−1^ within the reported bond wavenumbers, with no obvious differences in the types of bonds. For example, the broad hump and sharp peak in the regions of 3430–3460 cm^−1^ and 1020–1035 cm^−1^ still represent the stretching vibration of O-H and asymmetric stretching vibrations of Si-O-T (T = Si or Al) [9,12], but with a noticeable leftward shift, indicating the continuation of the polymerization reaction. The characteristics of the bands are summarized in Table 4.

#### 3.2.3. Microstructural Observation and Elemental Analysis

**Figure 10:**
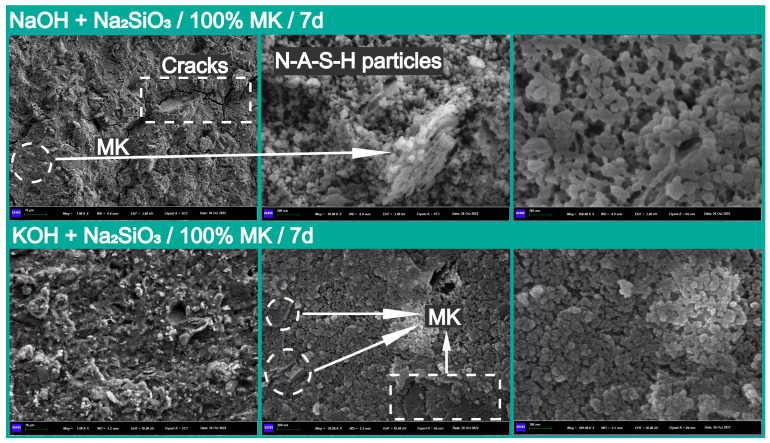
The microstructural characteristics of pure metakaolin-based geopolymers at 7 days.

**Figure 11:**
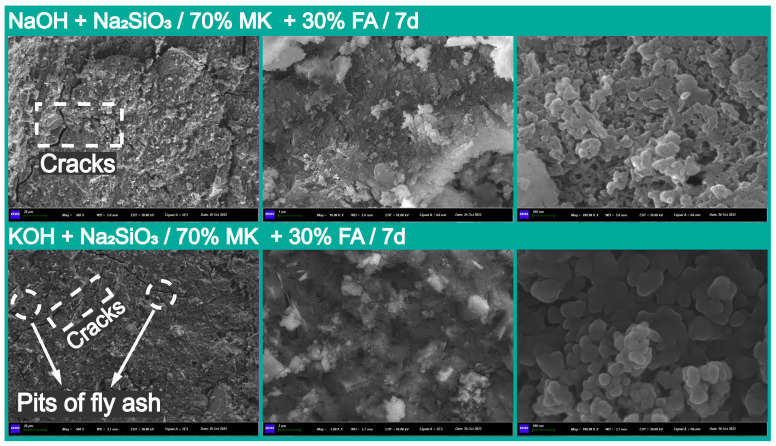
The SEM images analysis results for the No. 3 and 7 specimens with 70% metakaolin + 30% fly ash at 7 days.

To investigate the microstructure of the geopolymers with different cation types and fly ash content, the SEM image analysis results of gels, cracks, and partially reacted metakaolin in pure geopolymer-based geopolymer matrixes and 70% metakaolin + 30% fly ash geopolymers with the best compressive strength are provided in Figure 10 and Figure 11. It can be observed that the main product of the polymerization reaction was a dense polymer structure composed of N-A-S-H, exhibiting a typical granular morphology, while bonding the reacted flaked metakaolin into a homogeneous whole [20].

Additionally, the dense microstructure formed by the N-A-S-H particles significantly contributed to the early compressive strength at 7 days. In contrast, the incorporation of fly ash led to a more compact microstructure in the metakaolin and fly ash geopolymers compared to that observed in pure metakaolin-based geopolymers, suggesting a potential reason for their high compressive strength [59]. The formation of micro-cracks is mainly due to chemical shrinkage in the process of polymerization or mobile channels for water molecules, and significant affects the structural integrity and homogeneity of geopolymers [12], thus further deteriorating their mechanical and durability properties. Furthermore, the morphologies of (N, K)-A-S-H exhibit a high surface area with mesoporous intercommunicating mesh-like structures [60].

**Figure 12:**
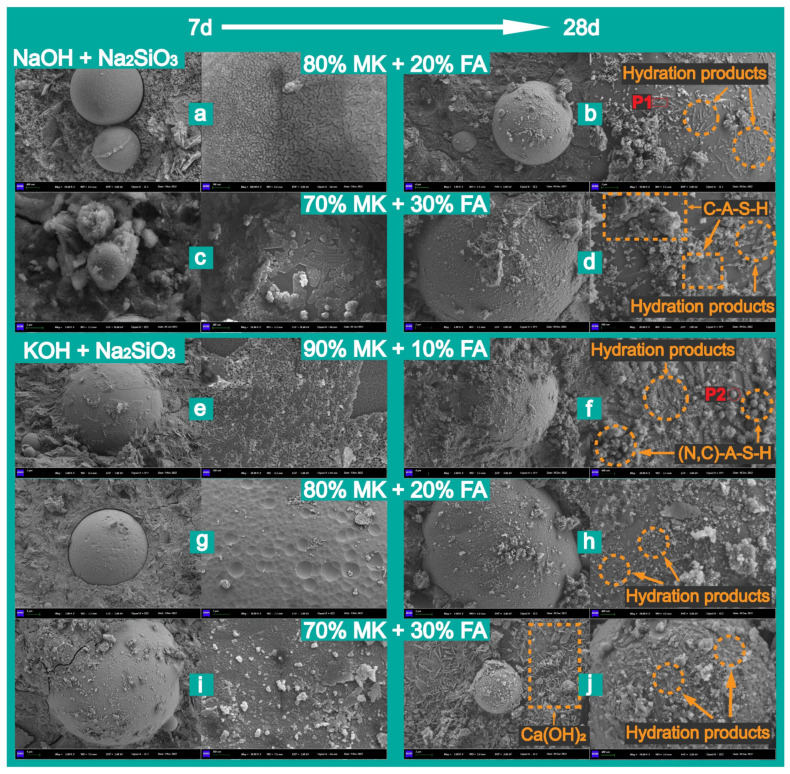
Hydration results and products of fly ash at 7 days and 28 days: (**a**) 80%MK + 20%FA at 7 days (NaOH + Na_2_SiO_3_), (**b**) 80%MK + 20%FA at 28 days (NaOH + Na_2_SiO_3_), (**c**) 70%MK + 30%FA at 7 days (NaOH + Na_2_SiO_3_), (**d**) 70%MK + 30%FA at 28 days (NaOH + Na_2_SiO_3_), (**e**) 90%MK + 10%FA at 7 days (KOH + Na_2_SiO_3_), (**f**) 90%MK + 10%FA at 28 days (KOH + Na_2_SiO_3_), (**g**) 80%MK + 20%FA at 7 days (KOH + Na_2_SiO_3_), (**h**) 80%MK + 20%FA at 28 days (KOH + Na_2_SiO_3_), (**i**) 70%MK + 30%FA at 28 days (KOH + Na_2_SiO_3_), (**j**) 70%MK + 30%FA at 28 days (KOH + Na_2_SiO_3_).

Additionally, the morphological characteristics of fly ash in the binary polymerization system in the geopolymer at different curing times are presented in Figure 12. Generally, the breaking process of the glass layer on the surface of fly ash in alkaline solution is significantly inhibited at lower temperatures. Therefore, the lower hydration rate of fly ash prolonged the time for the metakaolin–fly ash based geopolymer to achieve the maximum compressive strength. In contrast to the incompletely hydrated metakaolin particles, which were irregularly blocky as shown in Figure 10, the original unhydrated fly ash particles exhibited a spherical shape and were embedded in the matrix (Figure 12a). The action of the alkaline solution on the fly ash particles proceeded inward from the surface, with first eroding the glass layer on the fly ash surface, leaving numerous pits (Figure 12g). Subsequently, the glassy structure on the surface of the particles gradually disintegrated, resulting in lamellar silicate accumulations (Figure 12e,i). It could be clearly observed that chemical reactions begin to occur in the internal structure of fly ash, resulting in the formation of new products (Figure 12c). During the curing process from 7 to 28 days, the hydration products in the samples underwent significant evolution: in the NaOH-activated system containing 20% fly ash, the hydration gel gradually densified, and needle-like hydration products appeared on the surface of the fly ash particles, as shown in Figure 12c,d. In contrast, the samples activated with KOH exhibited the optimization effect of K^+^ on the aluminosilicate network structure, forming a more homogeneous (N,C)-A-S-H gel, as shown in Figure 12e,f. Notably, Ca(OH)_2_ present in the early stage was almost completely consumed by 28 days, likely due to its dissolution or transformation into other products in the highly alkaline environment. Overall, the extended curing time promoted the maturation of the gel products and the homogenization of the system. It was significantly noted that a large amount of sheet structure was embedded in the dense matrix, consisting of Ca(OH)_2_ crystals (CH), which could be the reason for the low compressive strength. CH interferes with the solidification process by reacting with dissolved substances or providing additional nucleation sites for dissolved monomers, while also reducing the alkalinity of the reaction system [61]. However, it should be noted that despite the presence of a limited proportion of calcium in fly ash, as indicated by the chemical analysis (Table 1), neither the crystalline peaks (Figure 6 and Figure 7) nor the FTIR spectra (Figure 8 and Figure 9) of the geopolymers indicated the formation of new C-(A)-S-H and CH phases. This may be due to the presence of soluble calcium material in the structure together with other components generating new amorphous hydration products [62]. In addition to the typical spongy C-A-S-H gel, a number of rod and spherical hydration products could also be observed on the surface of fly ash.

To further identify the hydration products observable on the fly ash surface, a portion of the fly ash, which was extensively distributed and could not be directly identified, was marked at specific points (P1 and P2) and subjected to EDS analysis. The results are shown in Table 5. It can be seen that at both locations, they consisted mainly of O, Al, and Si, regardless, which indicated the continuous formation of -O-Si-O-Al-O-Si-O- in the geopolymer network [9]. Moreover, the EDX spectra also clearly showed that the geopolymer gels, produced by the dissolution of the silicate and aluminate ions in the low-calcium fly ash, contained reaction products with low elemental Ca and Na contents, similar to the findings in the work of Khan et al. [63]. Regarding point 1, it is speculated that this might be the result of the aluminosilicate material within the fly ash beginning to leach out and combine with Na^+^ in the alkaline solution to form hydration products. In this process, there were two charge-balance pathways, namely, Na^+^ balance and the charge balance accomplished by the partial replacement of Na^+^ by Ca^2+^. This also explains why there was a 1.12% content of calcium atoms in the P2 point.

### 3.3. Solid Solution Reactivity and Inorganic Polymer Phase Evolution

Regarding the pure metakaolin-based geopolymer system, the typical hydration products included sodium aluminate silicate hydrate gels (Na_2_O-Al_2_O_3_-SiO_2_-H_2_O, N-A-S-H), which provides an important structural basis for the system’s properties (Figure 10). However, the XRD and FTIR analysis results indicated that the type of alkali cations did not significantly affect the evolution of the crystal phase and the basic structure (Figure 6, Figure 7, Figure 8 and Figure 9). The introduction of fly ash made the geopolymerization system more complex, which was mainly reflected in the types of hydration products. This could have occurred in stages, as illustrated below. However, it must be noted that the following description is based on the experiments performed in this study. By optimizing the mix, the precursor material used differs from that used by other researchers, and the instability of the material itself might have led to some inconsistencies with the results of other papers.

Step 1: dissolution of the precursor materials and release of monomers.

The first step of geo-polymerization involved the dissolution of aluminosilicate, which is significantly affected by the concentration of the alkaline solution [9]. In contrast to what observed with a low Si content and the insufficient formation of geopolymer gels caused by low alkalinity, high alkalinity resulted in a lower water content, making the alkaline solution highly viscous and the geopolymer paste less workable. Additionally, the geopolymer gels tended to precipitate prematurely. A similar suggestion was also proposed by Pascual et al. [64], who stated that ion mobility and stability are limited when the NaOH solution concentration exceeds 10 mol/L. In this work, the precursor materials (metakaolin and fly ash) released a large number of SiO_2_ and Al_2_O_3_ tetrahedral units in an alkaline environment. Then, these tetrahedral units were then bridged by shared oxygen atoms, resulting in gels with a three-dimensional mesh structure ((N, K)-A-S-H gels), predominantly derived from metakaolin (Equations (2)–(4)).

SiO_2_ (s) + Al_2_O_3_ (s) + 10OH^−^ (aq) → [SiO_2_(OH)_2_]^2−^ (aq) + 2[Al(OH)_4_ (aq) + 2H_2_O (aq) (2)

2Na^+^ (aq) + 4[SiO_2_(OH)_2_]^2−^ (aq) + 2[Al(OH)_4_]^−^ (aq) → Na_2_O·Al_2_O_3_·4SiO_2_·8H_2_O (gel) + 5OH^−^ (aq) (3)

2K^+^ (aq) + 4[SiO_2_(OH)_2_]^2−^ (aq) + 4[Al(OH)_4_]^−^ (aq) → K_2_O·Al_2_O_3_·4SiO_2_·11H_2_O (gel) + 9OH^−^ (aq) (4)

Regarding the alkali-activated system of metakaolin-based geopolymers, Alonso et al. [65] have stated that the gels in geopolymer systems can be classified into two main types, i.e., primary gels (N-A-S-H gels) and secondary gels (C-(A)-S-H gels) (Equation (5)), when the concentration of the alkaline solution is greater than or equals 10 mol/L. This finding is also consistent with the results in this study.

Al_2_O_3_ (s)+ SiO_2_ (s)+ Ca(OH)_2_ (s) + 2NaOH/KOH (aq) → (Na,K)_2_O·Al_2_O_3_·4SiO_2_·11H_2_O (gel) + CaO·Al_2_O_3_·2SiO_2_·4H_2_O (gel) + Unreacted phases (s) (5)

Meanwhile, it is important to note that the introduction of fly ash made these complex primary and secondary structures more evident during the hydration process of the calcium component. However, most research indicates that the beneficial effect of fly ash is primarily observed in the later hydration process, which aligns with our results. In the initial stages of the composite system’s reaction, the more chemically inactive glassy phases in fly ash, compared to metakaolin, allowed it to function as a structural filler embedded in the matrix (Figure 2) [66]. Therefore, the gel system in the early stage was mainly a N-A-S-H gel. However, this does not imply that the hydration reaction of fly ash completely ceased. It can be clearly noted in Figure 11 that the surface of the fly ash spheres was still slowly attacked and corroded by the alkaline solution in the system.

Step 2: water solubility of the calcium phase.

Actually, this step started to occur during the hardening of the wet material; however, due to the low dissolution rate of calcium oxide, the gel in the pre-ground polymer system was primarily dominated by N-A-S-H gels. As the degree of hydration of the system progressed, the spatial structure of fly ash disintegrated from the outside to the inside. CaO reacted with water to form calcium hydroxide (Ca(OH)_2_, CH) (Equation (6)), releasing a small amount of heat, which might create minor cracks in the structural system. Moreover, the presence of a large number of hydroxyl groups (OH^−^) in the system hindered the dissolution of CH (Equation (7)). Just a small amount of free Ca^2+^ existed in the channels of the system, making the precipitation of hydrated calcium silicate (C-S-H gels) difficult. The presence of large amounts of precipitated calcium hydroxide in geopolymer systems, where excess calcium-containing materials are present, hinders structural consolidation and strength development. A higher alkalinity of the dissolution medium, resulting from the reduction in water, favors the rapid dissolution of aluminosilicate and thus accelerate the rate of polycondensation–polymerization [67].

CaO (s) + H_2_O (l) → Ca(OH)_2_ (s) (6)

Ca(OH)_2_ (s) → Ca^2+^ (aq) + 2OH^−^ (aq) (7)

Step 3: formation of C-(A)-S-H gels and other hydration products.

As the number of OH^−^ ions in the system increased, immobilized in the (N, K)-A-S-H gels, subtle changes in the calcium hydroxide fraction began to occur. A portion of CH continued to precipitate and became enriched on the fly ash surface as well as elsewhere, along with the increasing hydration of fly ash. This was evidenced by the plate-like, hexagonal calcium hydroxide solids embedded in the matrix, as shown in Figure 12. Meanwhile, the other portion of the gradually dissolved Ca^2+^ and Ca^2+^ released from fly ash began to form richer hydration products with the various other ions present. For example, the increasing hydration of fly ash led to an effective replenishment of the alumina and silica content in the system, following their depletion in the first step. This promoted the formation of more silicate and aluminate species. In turn, calcium hydroxide provided additional nucleation sites for these free tetrahedral units to form new hydration products, such as calcium silicate hydrate (C-S-H) and calcium aluminate hydrate (C-A-H), as reported in the research analysis results of Du et al. [68] (Equations (8) and (9)). This process further optimized the pore structure, controlled nano-scale cracking, and improved the mechanical strength of the geopolymer [69].

Ca^2+^ (aq) + 2OH^−^ (aq) + SiO_2_ (s) → CaO·Al_2_O_3_·2SiO_2_·4H_2_O (gel) + H_2_O (l) (8)

Ca^2+^ (aq) + 2OH^−^ (aq) + Al_2_O_3_ (s) → CaO·Al_2_O_3_·2SiO_2_·4H_2_O (gel) (9)

Additionally, some of the aluminum ions (Al) were incorporated into C-S-H and formed C-A-S-H gels with a spongy appearance. Saloni et al. [70] pointed out that silicon atoms occasionally enter C-S-H gels but need to be adsorbed and combined with additional potassium or sodium ions to produce C-(N, K)-A-S-H, which was also one of the important hydration products [70].

### 3.4. Mechanism of Compressive Strength Development in the ‘Metakaolin + Low Calcium Fly Ash’ System

On the other hand, a notable result is that high values of 28-day compressive strength (up to 72.34 and 76.70 MPa for the NaOH/Na_2_SiO_3_ and KOH/Na_2_SiO_3_ specimens, respectively) were achieved at room temperature, without the need for heat curing during the early hardening stage, which has significant implications in terms of energy saving and environmental impact reduction. Based on the analysis results of XRD, FTIR, and SEM, the development of the geopolymer compressive strength in this system can be described considering two aspects: physical filling and chemical structure of the gels. The incomplete dissolution of chemically inert materials led to numerous pressure-bearing crystals filling the matrix. Some low-reactivity minerals (e.g., quartz, mullite, and anatase, Figure 6, Figure 7, Figure 8 and Figure 9) did not participate, or only partially participated, in the polymerization process acting as inset fillers. These were firmly embedded in the geopolymer matrix, enhancing the compressive strength. On the other hand, the complex gel structure generated sufficient internal stress to resist strong pressures while refining the internal pore structure, which was mainly due to the addition of calcium. The products of grade F fly ash in high-alkalinity solution were primarily geopolymer gels ((N,K)-A-S-H), with dissolved calcium ions contributing to the formation of C-(A)-S-H gel [19]. Additionally, García-Lodeiro et al. [71] suggested that the orientation or distribution of [SiO_4_] and [AlO_4_] seemed to be influenced by the presence of additional Ca, but this did not affect the 3D structure of N-A-S-H. Temuujin et al. [67] have previously demonstrated that calcium-containing compounds can effectively improve the mechanical properties and microstructure of fly ash-based polymers at ambient temperature; yet, the amount of calcium-containing compounds in the present experiments was minimal. However, it was difficult to detect calcium-containing crystalline phases other than CaCO_3_ using XRD, probably due to the formation of amorphous or poorly ordered crystalline forms. Nevertheless, the compressive strength resulting from the interaction between Ca(OH)_2_, derived from calcium-containing compounds, and aluminosilicate dissolved in an alkaline environment or Na_2_SiO_3_ is excellent [67].

## 4. Conclusions

This paper focused on preparing geopolymers from metakaolin and fly ash. The effects of fly ash levels and alkaline cation type on the compressive strength and microstructure of metakaolin–fly ash-based geopolymers were studied. In addition, the feasibility of preparing geopolymers at ambient temperature was preliminarily verified. Based on the above analysis, the following conclusions can be drawn:

(1) This study confirmed the feasibility of synthesizing high-compressive-strength composites using metakaolin and low-calcium fly ash at room temperature. The compressive strength of the geopolymer activated by NaOH reached 72.34 MPa. The compressive strength of the geopolymer activated by KOH was up to 76.70 MPa when the admixture of fly ash reached 20%.
(2) The increase in compressive strength can be attributed to the effective introduction of chemically inert fillers at the matrix interface and the formation of a dense, multidimensional gel structure. Among these inert fillers were mullite, which showed progressively sharper crystal peaks in the XRD spectra with increasing fly ash content, as well as the newly formed CaCO_3_. The key gel phases, including the granular N-A-S-H, spongy C-A-S-H, and needle-like C-S-H phases and other hydration products, played a role in this process. Additionally, the accelerated hydration of calcium ions and the provision of extra nucleation sites also contributed to the improved performance.
(3) Additionally, it should be noted that the type of alkaline activator did not appear to significantly affect the results in these experiments, as evidenced by the XRD and FTIR data. The combination of sodium hydroxide and sodium silicate remains the preferred choice due to its cost-effectiveness and minimal impact on compressive strength.

## Figures and Tables

**Table 1:** Chemical composition and particle size distribution of fly ash and metakaolin.

Chemical Composition	Quantity (% by Mass)	
	Fly Ash	Metakaolin
Al_2_O_3_	36.50	43.50
SiO_2_	43.70	47.90
CaO	5.19	0.86
Fe_2_O_3_	3.89	3.72
SO_3_	5.70	0.74
P_2_O_5_	1.63	0.41
TiO_2_	1.47	2.17
Others	1.92	0.70

**Table 2:** Particle size distribution of fly ash and metakaolin.

	D (10 nm)	D (50 nm)	D (90 nm)
Fly ash	302	357	422
Metakaolin	353	428	519

**Table 3:** Mixture parameters and compressive strength of geopolymer samples with two types of alkaline solutions, NaOH and KOH.

Mix No.	Replacement Rate of Fly Ash (%)	Mass Distribution (g)		Alkali Exciter (g)			Compressive Strength (MPa)	
		Metakaolin	Fly Ash	Na_2_SiO_3_	NaOH	KOH	7 Days	28 Days
1	0	1500	0	900	450	--	34.90	56.71
2	10	1350	150	900	450	--	38.73	67.26
3	20	1200	300	900	450	--	40.51	72.34
4	30	1050	450	900	450	--	37.24	61.09
5	0	1500	0	900	--	450	26.34	60.27
6	10	1350	150	900	--	450	30.92	70.89
7	20	1200	300	900	--	450	36.07	76.70
8	30	1050	450	900	--	450	27.41	60.21

**Table 4:** Characteristic FTIR bands of the geopolymer products.

Bands, cm^−1^	Assignments	References
3360–3460	Stretching vibration of O-H	[13,16]
1640–1660	Bending vibration of O-H	[13]
1053	Stretching vibration of Si-O-Si	[13]
1037	Asymmetric vibration of Si–O	[55]
970–1035	Asymmetric stretching vibrations of the Si-O-T (T = Si or Al)	[16,58,59]
865–870	The C-O-C bonds in the formation of calcite (CaCO3)	[52,60]
817	Symmetric stretching vibration of Si-O-Si bonds and stretching vibration of Al-O in mullite-like structures	[51]
670–685	The O-Si-O bands that correspond to the species of quartz	[58,61]
530–555	Symmetric stretching vibration of Al-O-Si	[52,59]
430–445	Bending vibration of Si-O-Si and O-Si-O	[53,54]
416	Bending vibrations of O-Si-O	[9]

**Table 5:** The analysis results of EDS at P1 and P2.

		O K	Na K	Al K	Si K	S K	K K	Ca K
P1	Weight%	53.71	5.69	17.52	20.35	0.04	2.36	0.33
	Atomic%	66.50	4.90	12.87	14.35	0.07	1.20	0.16
P2	Weight%	45.97	2.22	24.72	24.37	0.02	0.53	2.16
	Atomic%	59.59	2.00	19.00	17.99	0.07	0.28	1.12

## Data Availability

The original contributions presented in this study are included in the article. Further inquiries can be directed to the corresponding author(s).
